# Simulated large joint fluid model for evaluating intra-articular antibiotic delivery systems: initial evaluation using antibiotic-loaded calcium sulfate beads

**DOI:** 10.5194/jbji-7-117-2022

**Published:** 2022-05-17

**Authors:** Edward J. McPherson, Jessica A. Jennings, Omar Yunis, Michael A. Harris, Matthew V. Dipane, Nora L. Curtin, Madhav Chowdhry, Andrew J. Wassef, Joel D. Bumgardner, Scott P. Noel

**Affiliations:** 1 Department of Orthopaedic Surgery, David Geffen School of Medicine at UCLA, Santa Monica, 90404, USA; 2 Department of Biomedical Engineering, University of Memphis, Memphis, 38152, USA; 3 Long Beach Lakewood Orthopedic Institute, Long Beach, 90808, USA​​​​​​​; 4 Nuffield Department of Primary Care Health Sciences, Kellogg College, University of Oxford, Oxford, OX1 2JD, UK​​​​​​​; 5 Austin Medical Ventures, Germantown, 38138, USA

## Abstract

**Introduction**: Local antimicrobial delivery via calcium sulfate (CaSO ${}_{4}$ ) beads is used as an adjunctive treatment for periprosthetic joint infection. There is limited clinical information describing the performance of antimicrobial-loaded CaSO ${}_{4}$ (ALCS) in large-scale applications. We developed a simulated large joint model to study properties of eluting ALCS. **Methods**: The in vitro testing platform was an adapted standardized model for tribological testing of prosthetic total hips and total knees (ASTM F732). The model was 70 mL total fluid volume, 25 % bovine serum, and 75 % phosphate-buffered saline, using ISO standard 14242-1 for human synovial fluid simulation. Four brands of CaSO ${}_{4}$ were evaluated. Each 10 mL of CaSO ${}_{4}$ was loaded with 1.2 grams (g) of tobramycin and 1 g of vancomycin powders. A 35 mL bead volume, equaling 175 beads, of each product was placed in incubated flasks. The test period was 6 weeks with scheduled interval fluid exchanges. Fluid samples were tested for antibiotic and calcium concentrations and pH. **Results**: Antibiotic elution showed an initial burst on Day 1, followed by a logarithmic reduction over 1 week. Tobramycin fully eluted within 2.5 weeks. Vancomycin showed sustained release over 6 weeks. Calcium ion concentrations were high, with gradual decrease after 3 weeks. All four CaSO ${}_{4}$ products were inherently acidic. Fluid became more acidic with the addition of antibiotics primarily driven by vancomycin. **Discussion**: Clinicians should be cognizant of tobramycin elution burst with ALCS in large loads. The main driver of acidic pH levels was vancomycin. We propose that joint complications may result from lowered fluid acidity, and we suggest clinical study of synovial pH.

## 1 Introduction

Periprosthetic joint infection (PJI) is a debilitating complication of joint arthroplasty associated with higher mortality rates and healthcare costs than non-infected procedures (Kurtz et al., 2007, 2012). Treatment requires aggressive debridement with an implant exchange protocol (Maale et al., 2020b; Costerton et al., 1999; Hamad et al., 2022). Local antimicrobial delivery via dissolvable calcium sulfate (CaSO ${}_{4}$ ) using small beads (3–8 mm) has been utilized as an adjunctive treatment. The non-exothermic setting of CaSO ${}_{4}$ allows for almost all antimicrobial agents to be utilized (McPherson et al., 2021; Laycock et al., 2018). In PJI treatment, antibiotic-loaded CaSO ${}_{4}$ (ALCS) has been utilized since the late 1990s and ALCS application continues to evolve (Andreacchio et al., 2019; Kenna et al., 2018; Kurmis, 2021; Luo et al., 2016; Morley et al., 2022; Trujillo et al., 2017; Zhang et al., 2020; Ferguson et al., 2014; Abosala and Ali, 2020).

There is little clinical knowledge of the peri-articular elution kinetics using this delivery technique (Wahl et al., 2017). Confounding the problem, antimicrobial choice and concentrations within ALCS beads are surgeon-directed. Antimicrobial agents are used singularly or in combination with varying doses, limiting clinical analysis. In vitro models describe elution characteristics, but models vary and are generally of a small scale (Aiken et al., 2015; Bowyer and Cumberland, 1994; Grimsrud et al., 2011; Kanellakopoulou et al., 2009; Miclau et al., 1993; Moore et al., 2021; Roberts et al., 2014; Santschi and McGarvey, 2003; Wichelhaus et al., 2001). Treating clinicians are interested in ALCS elution characteristics simulating large-scale application.

We introduce a simulated large joint model (SLJM) to evaluate the properties of ALCS during antimicrobial elution. Our aim was to answer the key questions. What are the calcium ion levels with large bead volumes? How long are calcium ion levels elevated? What is the change in simulated joint fluid pH with ALCS bead elution? What are the antimicrobial elution kinetics using ALCS? Are there differences among CaSO ${}_{4}$ products? In this study, we measured fluid pH, calcium ion, and antibiotic concentration of eluate samples. Four (4) commonly used brands of CaSO ${}_{4}$ were tested. This study is designed to provide information to guide clinicians using ALCS in high bead volumes.

## 2 Methods

The SLJM testing platform was adapted from the standardized model used for tribological testing of prosthetic total hip and knee implants, as described by the ASTM F732. The sterile fluid media consisted of 25 % bovine serum (Gibco, Thermo Fisher Scientific, Waltham, MA) and 75 % phosphate-buffered saline (PBS), using ISO standard 14242-1, the accepted standard for human synovial fluid simulation (Bortel et al., 2015). The testing container was a 250 mL Pyrex glass container that was covered with a polyethylene seal cap. All preparations for the in vitro test platform were conducted under a vertical laminar flow hood. During the 6-week test, each container was placed on a LabDoctor Orbital Shaker (Midwest Scientific, St. Louis, MO) with gentle agitation (low setting). The initial preparation for each phase of the elution study consisted of 35 g of prepared CaSO ${}_{4}$ beads (calculated 35 mL bead volume) totalling 175 beads, added to 70 mL of fluid and placed within a closed 250 mL Pyrex glass container atop a continuous mixing platform.

Four brands of medical-grade CaSO ${}_{4}$ were evaluated. They were selected based upon FDA clearance for use in human subjects, commercial availability within the United States, and marketing as a bead kit. The selected products were Calcigen^®^ (Zimmer Biomet, Warsaw, IN), Osteoset™ (Wright Medical Group, Memphis, TN), Stimulan^®^ (Biocomposites, Staffordshire, England), and Synthecure^®^ (Austin Medical Ventures, Germantown, TN). Of the four, Calcigen and Osteoset are described as “mined and refined” products. These products are derived from mined gypsum that undergoes a multistep process to refine, wash, and sterilize the CaSO ${}_{4}$ , creating a powder meeting the ASTM standard for human medical use. Stimulan and Synthecure alternatively are synthetically derived products produced via a chemical manufacturing process. In general, these products are considered to be purer than their mined and refined counterparts (Liu, 2016).

**Figure 1:**
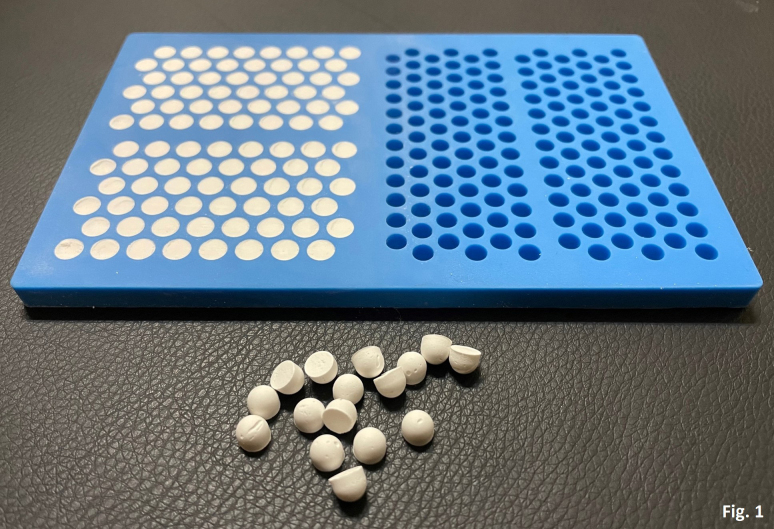
Picture of antibiotic-loaded CaSO ${}_{4}$ beads showing a 6 mm bead mat with cured CaSO ${}_{4}$ beads. Harvested beads next to the bead mat show the hemisphere shape of the beads.​​​​​​​

**Figure 2:**
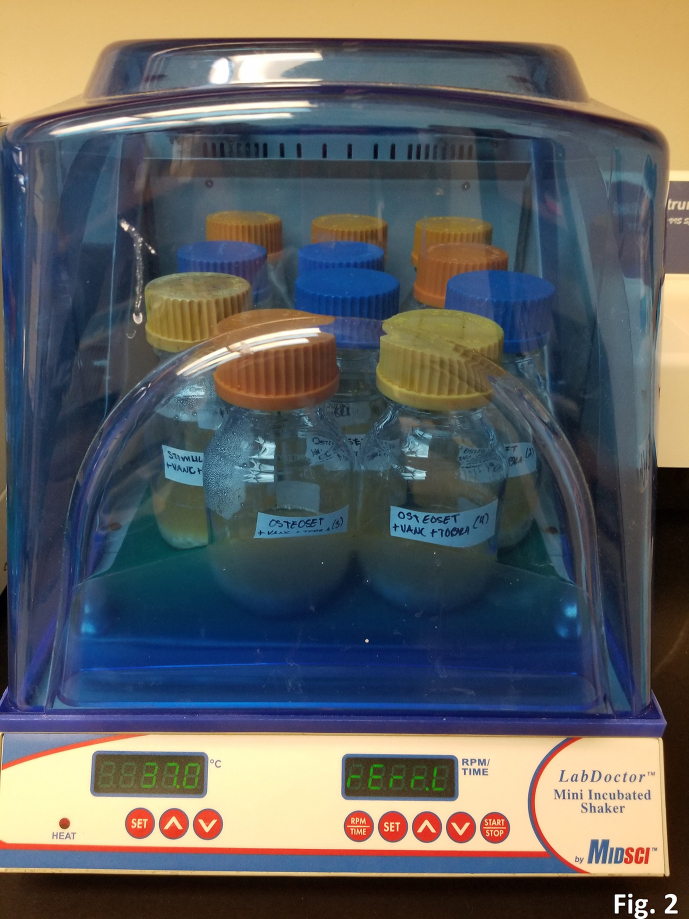
Simulated large joint fluid model setup. The 250 mL Pyrex container contains 70 mL of test fluid with 35 mL of CaSO ${}_{4}$ beads (totalling 175 beads) added to the fluid. The container sits atop a mixing stand stored in a sterile environment. The metal plate upon which the bottles rest is set on low shake to gently mix the fluid.

The CaSO ${}_{4}$ beads were prepared aseptically in a laminar flow hood. For each product, the 10 mL bead kits were used (10 mL defined as 10 mL of CaSO ${}_{4}$ paste after mixing). The antibiotic formula was 1.2 g of tobramycin powder (X-Gen Pharmaceuticals, Horseheads, NY) and 1 g of vancomycin powder (Breckenridge Pharmaceuticals, Berlin, CT) mixed into a 10 mL bead kit. The antibiotic powders were added to each of the four CaSO ${}_{4}$ products and hand-mixed aseptically prior to hydration. Each combined powder was hydrated with the included sterile hydration vial (H ${}_{2}$ O or saline, depending on the product) within the included plastic sterile mixing bowl until a fully homogenous paste was achieved. The paste was then spread into identical silicone bead molds (Synthecure molds) to produce 6.0 mm hemisphere beads (Fig. 1). The beads were left in the aseptic environment until cured. Bead setting was tested via bending of the mold. The CaSO ${}_{4}$ , once set, was harvested by bending the bead mold and allowing the beads to fall onto a sterile laboratory plate. Each 10 mL kit produces 25 mL in bead volume. For each test, 35 g of cured beads, equalling 35 mL in volume and totalling 175 beads (approximately 1.5 kits), were weighed aseptically on an analytical balance under the laminar flow hood and were subsequently added to the 70 mL fluid media. The test platform is shown in Fig. 2. Each product was tested four times and measured results were averaged. In addition, each product was tested four times without added antibiotics. Also tested were four control samples of simulation fluid (blank fluid) without beads.

Samples were taken during fluid refreshment at Days 1, 3, 7, 10, 14, 21, 28, 35, and 42. Under a sterile hood with aseptic technique, fluid was completely removed using a 50 mL pipette. The remaining beads were kept at the bottom of the container. Refreshment fluid was delivered into the container using a new 50 mL pipette. Tobramycin and vancomycin concentrations in each sample were measured using high-performance liquid chromatography (HPLC). Vancomycin concentrations were measured using absorbance at 209 nm. Measurements of tobramycin concentrations were derived using an automated pre-column method with 0-phthalaldehyde. Fluorescence was measured using an excitation of 337 nm and emission of 442 nm (Omar et al., 2015). Antibiotic activity within eluates was validated using a traditional Kirby–Bauer assay. Antibiotic activity of eluates (30 µ L) loaded onto 6 mm paper disks was evaluated against *Pseudomonas aeruginosa* and *Enterococcus faecalis*, susceptible to tobramycin and vancomycin, respectively. ImageJ was used to determine the zone for each sample. Calcium ion concentrations were measured using a colorimetric o-Cresolphthalein Complexone-based Calcium Reagent Kit (Pointe Scientific, Inc., Canton, MI), with absorbance measured at 600 nm. Acidity was measured using a standard benchtop pH meter. For the pH and calcium ion concentrations, the blank fluid medium was used as the negative control. The inherent pH values for tobramycin and vancomycin were tested separately from the test platform. Both tobramycin and vancomycin powders were reconstituted in their vials according to the manufacturers' directions. The resultant fluid was measured for pH and recorded.

## 3 Results

**Figure 3:**
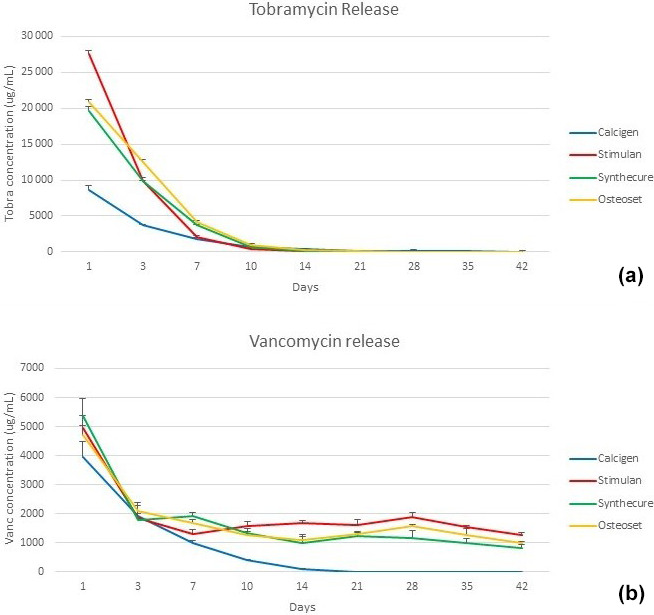
Tobramycin and vancomycin levels measured in the four CaSO ${}_{4}$ bead products. **(a)** A burst release of tobramycin was seen in all four products. The highest burst level was with Stimulan, the lowest with Calcigen. **(b)** The burst release of vancomycin was more muted (note that y -axis values are lower compared to panel **a**), and there was a long, steady-state release over the 6-week test interval. Bars indicate standard deviation.

Elution of the two antibiotics showed similar release patterns within the four tested CaSO ${}_{4}$ products and is shown in Fig. 3. In general, antibiotic elution demonstrated an initial burst release in the first 24 h, followed by a logarithmic reduction of antibiotics within 7 d. In this phase, tobramycin elution was much higher than that of vancomycin. In the burst phase, Stimulan showed a more pronounced release of tobramycin in the first 3 d compared to the other three products. Within the first 7 d, all products showed concentrations of vancomycin >1000 µ g/mL​​​​​​​, correlating with >100× minimum inhibitory concentration (MIC) to *E. faecalis*. During the same time period, tobramycin levels remained >1800 µ g/mL, correlating with >110× MIC to *P. aeruginosa*. Zones of inhibition confirmed antibiotic concentration and activity. After 7 d, antibiotic elution for both tobramycin and vancomycin demonstrated declining release kinetics. The extent of extended antibiotic release was greater with vancomycin. There was discernible vancomycin elution through the end of the 6-week test cycle, but the majority of the antibiotic was eluted within the first 4 weeks. Tobramycin elution was shorter, with a more abrupt exhaustion of antibiotic. The majority of tobramycin elution occurred within the first 2.5 weeks. Of the four CaSO ${}_{4}$ products tested, three products showed similar elution characteristics. The exception was Calcigen, where the burst release of tobramycin and vancomycin was substantially lower and there was an absence of steady-state release. The majority of antibiotic elution was completed far earlier than the other three products.

**Figure 4:**
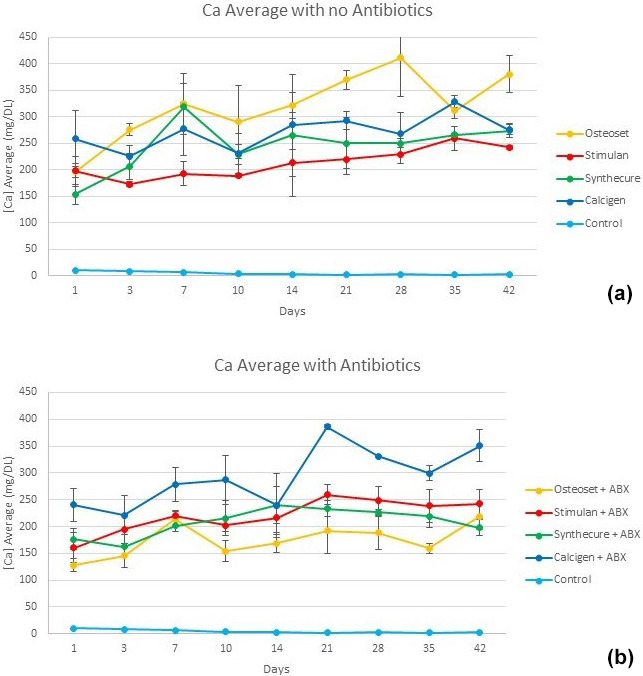
Calcium ion levels measured in the four CaSO ${}_{4}$ bead products with and without antibiotic loading. **(a)** With the exception of Osteoset, calcium ion levels rose slowly over the first 4–5 weeks as the unloaded beads dissolved. **(b)** Calcium levels are seen to decline after 3 weeks. There was more overall variability of measured values with Calcigen and Osteoset (mined and refined products). Bars indicate standard deviation.

Calcium ion release of the four products is shown in Fig. 4. There was no burst release of calcium as observed with antibiotic elution. In the unloaded state, calcium ion release started at a lower level and increased over 5 weeks as the beads dissolved. The exception was Osteoset. This product showed higher, more variable calcium ion concentrations over the entire test interval. Variations in calcium ion measurements were greater in the mined and refined products (Calcigen and Osteoset) compared to the synthetic products (Stimulan and Synthecure). In the antibiotic-loaded state, calcium levels slowly declined after 3 weeks. Compared to its unloaded state, calcium ion release in Osteoset was lower. With the synthetic products, calcium ion releases were similar in pattern and showed less variability. Toward the last week of the study, we observed crumbling/disintegration of the mined and refined products. This correlated with increased calcium ion concentrations in the tested fluid at the 6-week measurement. At the end of the 6-week test cycle, small residual bead products were still observable with Stimulan and Synthecure.

**Figure 5:**
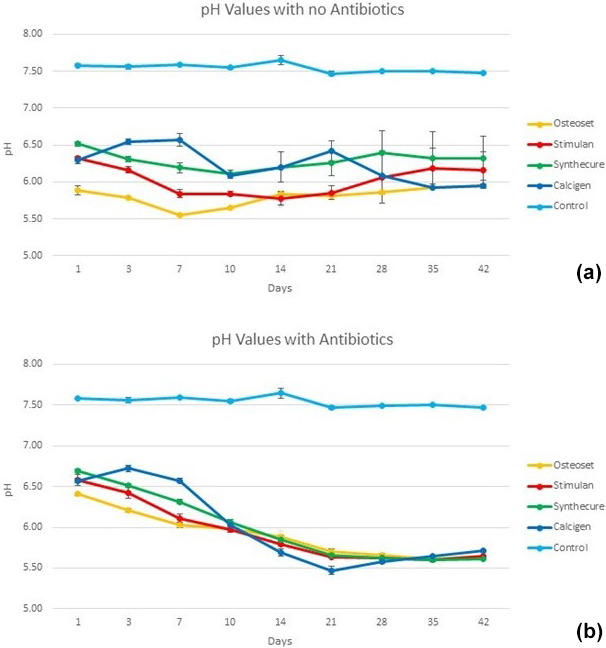
Fluid pH measurements in the four CaSO ${}_{4}$ bead products with and without antibiotic loading. **(a)** With the exception of Calcigen, there was an overall decrease in fluid pH values in the unloaded beads with a nadir between 7 and 10 d. The pH values slowly increased over the ensuing 4 weeks (again, the exception being Calcigen). **(b)** The fluid pH curves were dramatically different to their unloaded curves. Furthermore, all four products showed similar curve patterns, which we believe was driven by the combined inherent pH values of the antibiotics added to the CaSO ${}_{4}$ . Bars indicate standard deviation.

The 6-week pH values of the four CaSO ${}_{4}$ products are illustrated in Fig. 5. In the unloaded state (Fig. 5a), all four products were inherently acidic. The acidity curves for three of the products, Osteoset, Stimulan, and Synthecure, acted similarly. For these three products, pH values began acidic and decreased, with a pH nadir at 7–10 d, before gradually increasing back towards initial Day 1 levels. Calcigen, on the other hand, showed decreased acidity in the first 7 d and then became increasingly acidic over the following 5 weeks. Overall, the most acidic unloaded product was Osteoset. The least acidic product over the 6-week test was Synthecure.

The pH curves showed distinct changes when loaded with antibiotics (Fig. 5b). With antibiotic loading of tobramycin and vancomycin, all products, including Calcigen, showed similar pH curves. All four products remained acidic throughout the 6-week test. They each showed a gradual decline in pH values, converging towards a pH value near 6 at Day 10. Thereafter, there was a decrease in pH with a nadir at Week 4. All pH values of the four tested products remained more acidic at the end of 6 weeks compared to Day 1 pH measurements. The measured pH of reconstituted tobramycin was 7.2. The measured pH of reconstituted vancomycin was 3.5.

## 4 Discussion

For PJI treatment, ALCS has been utilized as an adjunctive treatment combined with implant exchange, radical debridement, and antibiotic-loaded acrylic cement (Abosala and Ali, 2020; Maale et al., 2020b; Malone et al., 2017; McPherson et al., 2013). Strategically, clinicians use ALCS beads with the premise that local delivery can potentially achieve antimicrobial gradients high enough to kill microbiota throughout the PJI space, including remnant biofilm (Maale et al., 2020a; Brooks et al., 2021; Moore et al., 2021). Laboratory models suggest ALCS beads can deliver antimicrobial concentrations that can eradicate microbes (including variant reserves) within a biofilm (Hamad et al., 2022; Sindeldecker et al., 2020; Sindeldecker and Stoodley, 2021). To achieve high intra-articular antibiotic levels, clinicians are inserting large bead volumes (50–150 mL), but there is a lack of information reporting elution kinetics of ALCS beads at these volumes. Previous in vitro elution models do provide helpful information but are smaller in bead volume compared to current in vivo applications and may not reflect clinical elution kinetics (Aiken et al., 2015; Bowyer and Cumberland, 1994; Grimsrud et al., 2011; Kanellakopoulou et al., 2009; Miclau et al., 1993; Moore et al., 2021; Roberts et al., 2014; Santschi and McGarvey, 2003; Wichelhaus et al., 2001). This model, we believe, comes closer to portraying elution kinetics of ALCS beads on the scales employed clinically. First, the testing medium simulates human synovial joint fluid following ISO standard 14242-1, as opposed to sterile water or saline. Using this standard, fluid protein concentration and pH simulate human synovial fluid, both of which affect fluid antibiotic and calcium ion concentrations. Second, the fluid was continually mixed and underwent exchange on a repetitive basis to simulate joint fluid dynamics. A pure static model, without fluid mixing and exchange, does not accurately reflect joint fluid rheology. To recreate joint fluid flow, an ideal model would require continuous fluid exchange combined with intermittent pauses of mixing. The compromise we chose, for economics and “best realism”, was a gentle mixing with scheduled fluid exchanges. Lastly, the bead volume to fluid volume ratio was close to proportions utilized in clinical hip and knee joint cases. We believe the testing of 175 beads in 70 mL of fluid is more reflective of clinical ALCS use.

Our model revealed interesting findings. First, antibiotic elution curves showed similar patterns. There was an initial burst of tobramycin and vancomycin on Day 1. We ascribe this high peak effect to the release of the two antibiotics from the superficial surfaces of the CaSO ${}_{4}$ beads. The release logarithmically declined over a period of 5–7 d to reach a steady-state release at a lower rate. In all four ALCS products, vancomycin release showed a peak delivery and then rapidly diminished over 7 d to release thereafter at a steady state. Vancomycin elution then remained at a lower rate over a period of 5 weeks. In contrast, tobramycin release was more rapid. A higher tobramycin burst release was observed, with a more rapid decline within the first 7 d. Its steady-state release was abrupt, with complete elution achieved within 2.5 weeks. We describe tobramycin release as an “antibiotic dump.” The early antibiotic bursts seen in this test platform have clinical relevance. The high peak values seen in the burst phase may cause detectable serum levels of these antibiotics via bead delivery alone. Clinicians managing parenteral antibiotics should be aware of this burst phenomenon. Until there is well-established clinical correlation of these findings, we suggest an early postoperative measurement of serum tobramycin and vancomycin before starting customary loading doses of parenteral antibiotics that are potentially nephrotoxic (Wahl et al., 2017). We also suggest clinicians be cognizant of administering other nephrotoxic agents and advise maintaining euvolemic hydration and monitoring renal function carefully within the first 10–14 d of treatment.

**Figure 6:**
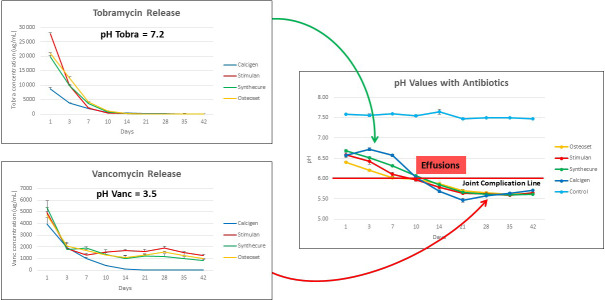
Visual display of the proposed joint complication line, showing the influence of tobramycin and vancomycin upon net fluid acidity. In this model, tobramycin buffered fluid pH until it was depleted. Thereafter, the low pH of vancomycin took over to reduce fluid pH below a critical value (estimated here as an example of pH 6.0) that we name the “joint complication line.” When pH values drop below this theoretical value, joint complications we propose are more likely to occur.

Calcium ion levels of ALCS did not show a burst effect. Instead, there was a gradual rise of fluid calcium ion concentrations in the first 3 weeks, after which there was a levelling and gradual reduction. The curve patterns were steadier with the synthetically derived products. We attribute the rising calcium ion concentrations to the ongoing dissolution of the CaSO ${}_{4}$ beads. As a bead degrades, the surface area of the bead product increases. This increased surface area to volume ratio provides greater calcium ion release. After 3 weeks, calcium ion levels slowly decreased, which we ascribe to the progressive reduction in bead size. With mined and refined products, we ascribe the late increase in calcium ion concentrations to bead disintegration. We observed that mined and refined products were not as consistent in mixing and plating compared to the synthetic products. This is due to the inherent variability of gypsum composition, which can adversely affect bead crystallization. Finally, the high calcium levels seen in all four products corroborate clinical observations of serum hypercalcemia with high intra-articular bead loads (Kallala and Haddad, 2015; Kalalla et al., 2018; Kuo et al., 2019). We suggest future study to modify CaSO ${}_{4}$ beads to dissolve with lower peak calcium ion levels and elute antimicrobial agents with modest burst effects.

The pH measurements of CaSO ${}_{4}$ beads provide interesting insight. In the unloaded state (not utilized clinically in PJI), pH levels were acidic overall. Of the two synthetic products, Synthecure had higher pH values than Stimulan. We assert that the “chemical source products” used to manufacture CaSO ${}_{4}$ do influence inherent pH to some extent. Interestingly, in the loaded scenario, all products performed comparably and demonstrated dramatically different pH curves to their unloaded states. The pH values of the loaded products declined after 10–14 d, with all four products demonstrating very similar pH curve profiles. It is our contention that the lower pH values observed result from the addition of antibiotics to CaSO ${}_{4}$ , creating a resultant net acidity. We acknowledge that the pH buffering capacity of the human joint may better mitigate the observed changes seen in this model and this will be a future area of clinical study.

The combined graphs in Fig. 6 show the antibiotic effects on net pH in the test fluid, which we believe to have potential clinical relevance. First, tobramycin (pH 7.2) releases rapidly within the first 14–18 d. We contend that this serves to buffer the effect of the more acidic vancomycin (pH 3.5). Once tobramycin is depleted, acidity increases due to prolonged vancomycin elution. In our clinical observations of over 1400 ALCS bead cases, we have observed wound drainage and joint effusions occurring late in the second week post-operative with bead volumes ≥20 mL. Other reports in the literature observe wound drainage in relation to ALCS use without specifying temporal presentation (Kallala et al., 2018​​​​​​​; Abosala and Ali, 2020). This correlates with this model when the net fluid pH drops below 6.0. Hence, we propose the concept of the “joint complication line.” We speculate that joint effusions and wound leaks occur, not primarily by increased oncotic joint pressure from CaSO ${}_{4}$ dissolution, but rather when joint fluid pH drops below “a critical” complication line which has yet to be clinically defined.

This proposed mechanism for joint effusion and wound leaks is in no way established by this study, but this model does, at a minimum, provide a basis for exploring the clinical kinetics involved in CaSO ${}_{4}$ dissolution and antibiotic elution. Going forward, we aim to next establish acidity curves for ALCS beads, testing antibiotics individually. By studying pH curves, combination antibiotic therapy could, in the future, be tailored to avoid lowering net joint fluid pH below a determined (future study) joint complication line.

In summary, this SLJM was developed to better understand the elution kinetics of ALCS beads in a large-scale simulation of a human joint. With this first iteration, we have established preliminary elution curves that include tobramycin, vancomycin, calcium, and fluid pH. Vancomycin elutes, in general, more gradually than tobramycin. Inherent CaSO ${}_{4}$ pH levels are acidic in simulated fluid media. With antibiotic loading, we ascribe the unique pH curves to the differences in antibiotic elution over time. The most relevant data were elicited within the first 4 weeks. Our model also demonstrated more predictable elution curves of synthetic products when compared to mined and refined products. Going forward, our next iteration of testing will be conducted over a period of 4 weeks with more frequent fluid exchanges and testing intervals. We also plan to incorporate shaking rest periods to simulate joint rest. We plan to validate this model with data obtained from clinical applications of ALCS. Our goal is to establish this test platform as a screening model for future delivery agents utilizing various antimicrobial medications.

## Data Availability

This study does not have any underlying research data. All data is presented in the manuscript in graphical format.
